# Dormancy-release, germination and seedling growth of *Paeonia ostii* ‘Fengdan’ seeds under measures of physical and chemical treatment and sowing

**DOI:** 10.1371/journal.pone.0270767

**Published:** 2022-07-05

**Authors:** Yuying Li, Qi Guo, Kaiyue Zhang, Hao Wang, Changsong Jia, Dalong Guo, Lili Guo, Xiaogai Hou

**Affiliations:** 1 College of Agronomy/College of Tree Peony, Henan University of Science and Technology, Luoyang, Henan, China; 2 Beijing Changsong Technology Co., Ltd, Beijing, China; 3 College of Forestry, Henan University of Science and Technology, Luoyang, Henan, China; Department of Agronomy, University of Agriculture, Faisalabad, PAKISTAN

## Abstract

*Paeonia ostii* ‘Fengdan’, a woody oleaginous plant native from China, is considered an oil crop with economic potential. However, a low germination rate was still a restriction for *Paeonia ostii* ‘Fengdan’ production. The present research evaluated the germination, rooting and physiological characteristics of seedlings of *Paeonia ostii* ‘Fengdan’ in response to different physical treatments and the application of exogenous chemicals. Results indicated that seeds stored in sand at room temperature, and soaked in water for 3 days prior to planting, had a beneficial effect on hypocotyl dormancy-breaking. The rate of rooting and root growth of *Paeonia ostii* ‘Fengdan’ were significantly improved with 5 cm sowing depth in 15–20℃ soils. Compared with other sowing depths, the rooting percentage was significantly increased by 1.19% (2.5 cm), 0.98% (7.5 cm) and 1.47% (10 cm), respectively. Epicotyl dormancy was relieved when taproot length reached 50 mm. Soaking seeds in 0.76 mmol/L 5-aminolevulinic acid for 48 hours had the greatest beneficial effect on seed germination and seedling growth, the germination percentage was significantly increased by 4.25% (24 h) and 5.08% (72 h) compared with other treatments. While seed soaked in 10 mmol/L sodium nitroprusside for 48 hours also exhibited enhanced seedling growth, and the germination percentage was significantly increased by 4.36% (24 h) and 7.40% (72 h). Those results benefited seed germination and seedling growth of *Paeonia ostii* ‘Fengdan’ which could suggest the promotion of its industrial values and productive potentials. The mechanism of seed breaking dormancy and germination of *Paeonia ostii* ‘Fengdan’ needs further study.

## 1 Introduction

Tree peony (*Paeonia suffruticosa*) is a perennial deciduous shrub in the section *Moutan* DC. of the genus *Paeonia* L. (Paeoniaceae) and is endemic to China [1]. The oil extracted from tree peony seeds represents an important edible oil in China due to its high content of unsaturated fatty acids (UFAs) (over 90%) with over 40% linolenic acid (ALA) [2, 3], and the market for tree peony seed oil has been expanding. Consequently, the planting area dedicated to oil tree peony has been increasing every year. In 2011, the Ministry of Health of the People’s Republic of China recognized oil tree peony as a new woody grain and oil plant resource [4]. The planting area of oil tree peony in China is expected to reach more than two hundred thousand hectares in 5–10 years [5]. According to statistics, the seed yield of oil tree peony is 3750 kg/ha. This production scale could result in 750 thousand tons of seeds per year [5, 6]. At present, *Paeonia ostii* ‘Fengdan’ is the main cultivated species of oil tree peony and cultivar planted due to its superior seed and oil yield [7].

Seed dormancy is an adaptive property of plants to avoid and/or resist the long-term adverse growth conditions, which could regulate the optimal time and spatial distribution of seed germination [8]. Breaking seed dormancy is an indispensable requirement for commercial growing crops which is genetically determined and dependent to the seed’s structure and growth ability of the embryo [9]. Dormancy-breakingis also regulated by environmental conditions and endogenous hormones [10, 11]. Most researches on seed dormancy-breaking and germination focused on improving seed germination by chemicals [12, 13] such as GA_3_ [14, 15], abscisic acid (ABA) [16, 17], and sulfuric acid [18, 19].

Seed germination is critical factor in crop production [20]. Traditional propagation of *P*. *ostii* ‘Fengdan’ plants has been typically done through seed propagation. Germination rates under natural conditions, however, are low and require extended periods of time, while the survival percentage is also low [21]. Seeds of *P*. *ostii* ‘Fengdan’ also exhibit characteristics of double dormancy and physiological post-ripening [22]. Natural matured seeds of *P*. *ostii* ‘Fengdan’ need to mature for a period of time to overcome hypocotyl dormancy, and epicotyl dormancy is overcome only after rooting has progressed to a certain stage [23, 24]. In addition, the binding force as mechanical barrier between the seed coat and endosperm also limited the dormancy-breaking of *P*. *ostii* ‘Fengdan’ seeds. Therefore, mechanical peeling of the seed coat has been used to increase the permeability of the seed shell and promote seed germination in *P*. *ostii* ‘Fengdan’ [25].

Recently, studies on *P*. *ostii* ‘Fengdan’ focused on container culture and indoor seedling cultivation [26], dormancy-breaking by low-temperature exposure [27], and the use of plant growth regulators (PGRs) [28–30]. However, studies on the effect of sowing measures and the use of exogenous chemical substances on dormancy-breaking, germination and seedling growth in *P*. *ostii* ‘Fengdan’ have not been explored. Therefore, the present research evaluated different methods that could be used to increase germination in *P*. *ostii* ‘Fengdan’ and improve its potential use as an oil-seed crop.

## 2 Materials and methods

### 2.1 Study site and materials

**Fig 1:**
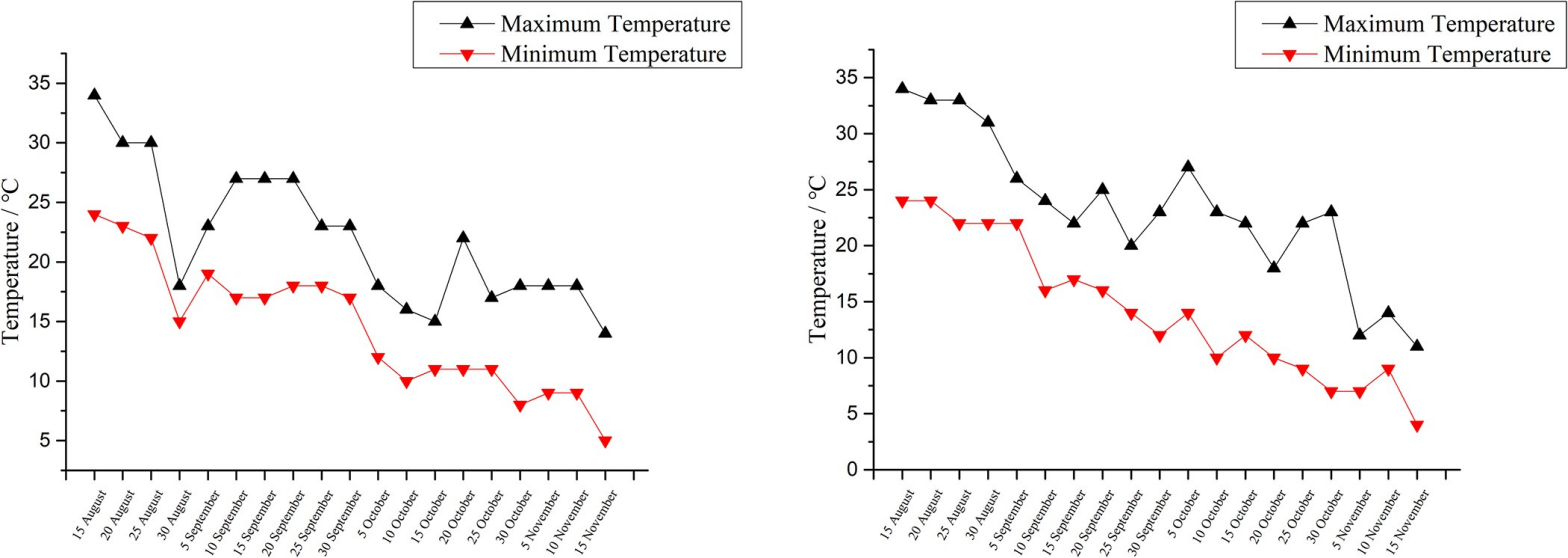
Daily temperature variation map of the test area. A: 2017; B: 2018.

The experiments were conducted on the experimental farm of Henan University of Science and Technology, Luoyang, China (112°36’19.65" E, 34°39’55.43" N). This area has a temperate, semi-humid, semi-arid, continental monsoon climate with an average annual rainfall of 600 mm and an average annual temperature of 12.1–14.6℃. The average temperature in January was 0℃ and 27℃ in July. The average annual radiation was 491.5 kJ/cm^2^, the annual sunshine was 2300–2600 h, and the frost-free period was 215–219 d. The soil texture is sticky and is either neutral or slightly alkaline. The whole soil profile is free of calcium carbonate, containing a small amount of calcium oxide, low level of organic matter (13.18 g/kg), and a salt base saturation ≥ 80%. In addition, there are 0.79g/kg total nitrogen, 74.21 mg/kg alkali-hydrolyzed nitrogen, 11.26 mg/kg available phosphorus and 147.80 mg/kg available potassium in 0–40 cm soil layer. The daily temperatures during the experiment are shown in Fig 1.

**Fig 2:**
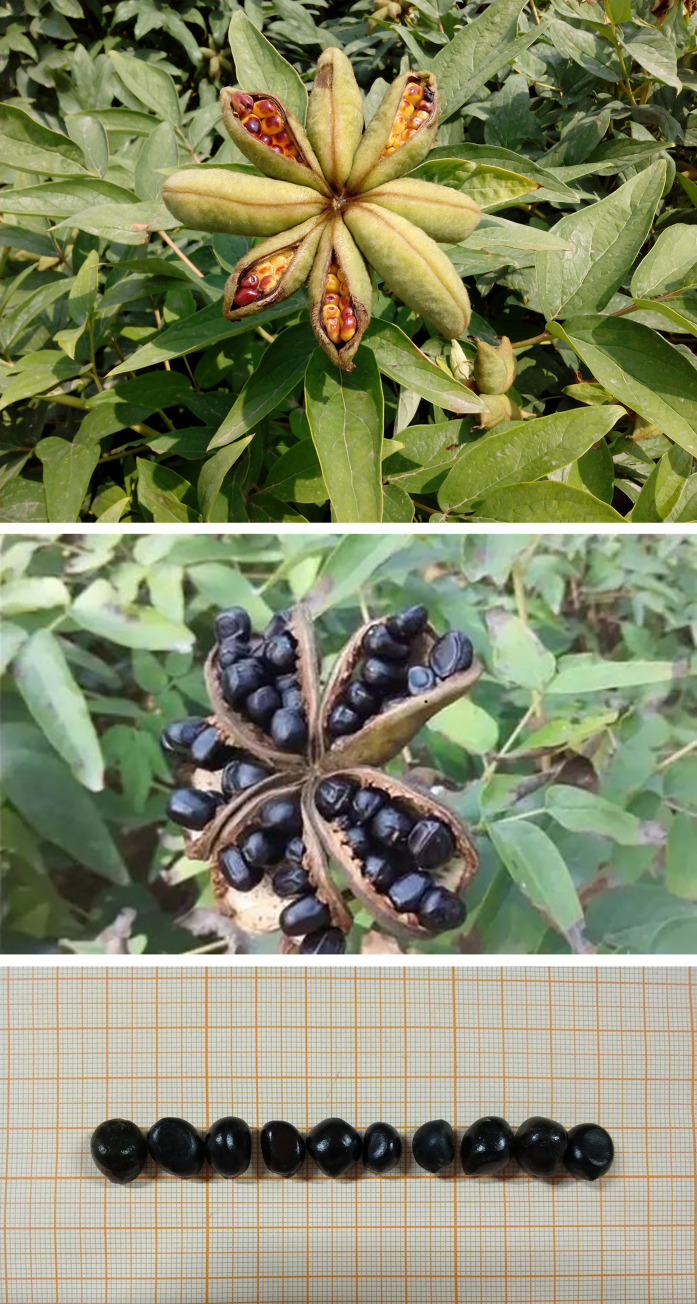
The ripening process of *P*. *ositt* ‘Fengdan’. A: Tree peony fruit; B: The fruit after the completion of ripening; C: Mature seed.

Seeds were collected from fresh fruits of five-year-old uniformly and well grown *P*. *ostii* ‘Fengdan’ plants. The fresh fruits were collected during the standard harvest period when the fruit peels turn crab yellow (Fig 2A), and were spread out indoors for 3–5 days to mature naturally. Seeds were collected after the fruit peel had cracked (Fig 2B and 2C). Then the collected seeds were placed in water and unfilled seeds (floating seeds) were removed so that only full seeds were used in the planned experiments. The selected seeds were soaked in 0.5% KMnO_4_ for 2 h and then rinsed by sterile water for 3–5 times to disinfect.

### 2.2 Experimental design

The disinfected seeds were treated by three seed treatment approaches (sand storage, seed soaking duration and chemical treatment) and two sowing approaches (sowing date and sowing depth). Non-treated seeds were used as a control. Experiments were conducted between August, 2017 and November, 2018.

#### 2.2.1 Sand storage duration

Selected seeds were soaked in distilled water for 24 h and then placed in high-temperature sterilized (121℃) river sand with seeds to sand volume ratio of 1:3. The seeds were stored in sand at room temperature (20–25℃) for 15, 30, 45, 60 days. After sand storage, seeds were sown in 10 cm containers filled with a mixture of peat soil, humus, and vermiculite powder (1:1:3 v/v) on September 30, 2017. In each container was sown one seed, and then buried in the field. Each treatment comprised 100 seeds replicated three times.

#### 2.2.2 Seed soaking duration

Selected seeds were soaked in distilled water for 0, 3, 5, 7 days at room temperature. The seeds were then sown in 10 cm containers filled with a mixture of peat soil, humus, and vermiculite powder (1:1:3 v/v) on August 30, September 15, September 30, October 15, and October 30, 2017. In each container was sown one seed, and then the containers were buried in the field. Each treatment comprised 100 seeds replicated three times.

#### 2.2.3 Chemical treatment

Selected seeds were soaked in different concentrations (0.38 mmol/L, 0.76 mmol/L, 1.52 mmol/L and 3.04 mmol/L) of 5-aminolevulinic acid (5-ALA) or sodium nitroprusside (SNP) (5 mmol/L, 10 mmol/L, 15 mmol/L and 20 mmol/L) for 24, 48, or 72 h. The seeds were then washed with distilled water and sown in the field on October 10, 2018. The experimental field had been ploughed to a depth of 20 cm and furrows were established for sowing. Each treatment comprised 100 seeds replicated three times.

#### 2.2.4 Sowing date

Selected seeds were first soaked in distilled water for 3 days and then sown in the field on September 10, September 20, September 30, October 10, October 20, and October 30, 2018. The experimental field had been ploughed to a depth of 20 cm and furrows were established for sowing. Each treatment comprised 100 seeds and each treatment was replicated three times.

#### 2.2.5 Sowing depth

Selected seeds were first soaked in distilled water for 3 days and then sown in the field at a depth of 2.5, 5.0, 7.5, or 10.0 cm on September 10, September 20, September 30, October 10, October 20, and October 30, 2018. Air and ground temperatures were recorded. The experimental field had been ploughed to a depth of 20 cm and furrows were established for sowing. Each treatment comprised 100 seeds and each treatment was replicated three times.

### 2.3 Measured parameters

Ground temperature at soil depths of 5, 10, 15, and 20 cm were measured every 10 days at 19:00 pm using a WQG-16 curved tube geometer. The initiation of rooting, rooting percentage, and root length were evaluated every 7 days beginning 10 days after the seeds were sown. The percentage of roots longer than 50 mm was recorded at 90 days after sowing. Root morphology was recorded with the aid of a root scanner (Epson Expression 1680 Scanner, Seiko Epson Cor*Paeonia*, Tokyo, Japan), and analyzed using WinRHIZO root system (Regent Instruments Inc., Quebec, Canada) software. Seeds germination percentage, seedling height, leaf length, leaf width and the number of leaves were in each treatment were all recorded. The calculation formula is as follows:

Rooting percentage % = (L1/L0) × 100%

Rooting percentage of root length ≥ 50 mm % = (L2/L0) × 100%

Germination percentage % = (L3/L1) × 100%

Where, L1 is the number of rooting seeds in experimental seeds, taking radicle breakthrough through seed coat as the standard. L2 is the number of root seeds whose root length is ≥ 5cm. L3 is the number of germinated seeds in experimental seeds. L0 is the number of experimental seeds.

Leaves of seedlings derived from the treated seeds were harvested in June 2018, washed with distilled water, immediately frozen in liquid nitrogen and stored in the -80℃ refrigerator. Soluble sugar content, malondialdehyde (MDA) content, and proline content in leaves obtained from the treated plants were determined spectrophotometrically using anthrone sulfate [31], the thiobarbituric acid method [32], and the ninhydrin-sulphosalicylic acid method [33] respectively.

### 2.4 Statistical analysis

The significance of treatment effects was determined by ANOVA in SPSS 21.0. Significant differences between the treatment groups were determined using a Duncan’s new multiple range test (*P* < 0.05). The results were plotted using Origin 8.5 software.

## 3 Results

### 3.1 Effect of sand storage duration on rooting and germination of *P*. *ostii* ‘Fengdan’

Proper sand storage treatment can promote the rooting of *P*. *ostii* ‘Fengdan’, shorten the rooting time and increase the rooting rate. Hypocotyl dormancy-release in *P*. *ostii* ‘Fengdan’ seeds stored in sand at 20–25℃ for 30 days occurred 26 days earlier than it did in the control seeds (Table 1). Increasing the length of sand storage duration did not further shorten hypocotyl dormancy as the time to rooting remained relatively stable. The rooting and germination percentage of seeds with a taproot length ≥ 50 mm gradually increased with sand storage duration. The rooting percentage (95.83±3.33%) and the germination percentage (78.00±9.25%) reached their optimum after 60 days of sand storage. Compared with other treatments, the germination percentage of *P*. *ostii* ‘Fengdan’ was improved significantly by 3.00–62.16% and 3.33–76.83%, indicating that sand storage improved the quality of rooting.

**Table 1:** Effect of sand storage duration on rooting and germination of *P*. *ostii* ‘Feng Dan’.

Sand storage duration (d)	First rooting days (d)	Rooting percentage (%)	Germination percentage (%)	Rooting percentage of taproot length ≥50 mm (%)	Germination percentage of taproot length ≥50 mm (%)
0	44	33.67±0.60 d	53.33±2.74 b	0.00±0.00 c	0.00±0.00 b
15	43	87.00±1.32 c	73.50±11.81 a	62.83±2.31 b	78.00±8.37 a
30	18	89.17±3.51 bc	76.50±13.65 a	66.33±1.26 b	82.00±4.47 a
45	18	92.83±2.36 ab	77.00±8.37 a	73.50±3.46 a	82.00±4.47 a
60	18	95.83±3.33 a	78.00±9.25 a	76.83±5.06 a	82.00±8.37 a

Note: Different lowercase letters indicate that the significant difference between each treatment at 0.05 level.

**Fig 3:**
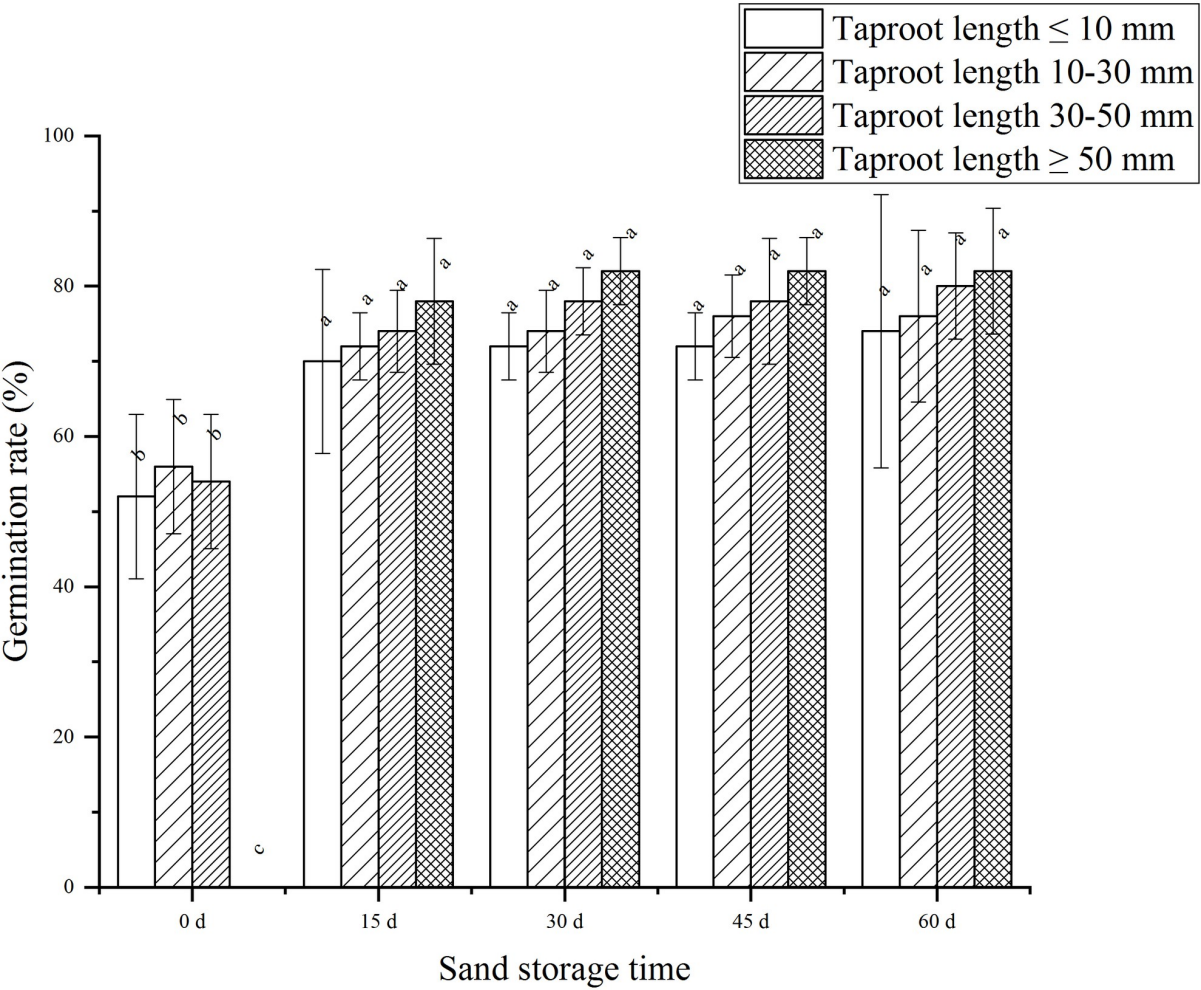
Effect of different root lengths on germination of *P*. *ositt* ‘Fengdan’ seeds.

Results also indicated that epicotyl dormancy-release in *P*. *ostii* ‘Fengdan’ seeds was directly related to root length (Fig 3). Seed germination increased with taproot length, indicating that the germination of seeds was affected by the extent of rooting. Combined with the influence of taproot length on germination percentage of sand storage treatment 30 d-60 d, the results showed that the germination percentage of *P*. *ostii* ‘Fengdan’ under taproot length ≥ 50 mm was 11.38% (taproot length ≤ 10 mm), 8.13% (taproot length 10–30 mm) and 4.07% (taproot length 30–50 mm) higher than that under other taproot length conditions, respectively. In addition, too long radicle is not conducive to practical production measures. There was no significant difference in rooting percentage (92.83±2.36%), germination percentage (77.00±8.37%), rooting percentage (73.50±8.37%) and germination percentage (82.00±4.47%) of taproot length ≥ 50 mm after sand storage duration for 45 d and 60 d. Thus, the sand storage duration of 45 days appears to be most appropriate.

### 3.2 Effect of soaking duration on germination and seedling growth of *P*. *ostii ‘*Fengdan’

Suitable soaking time can effectively promote the lifting of hypocotyl dormancy of *P*. *ostii* ‘Fengdan’ (Table 2). Germination percentage, seedling height, leaf length and leaf width initially increased with increasing soaking duration but then decreased. All of the measured indices reached a maximum value at 3 days of soaking, and the germination rate of *P*. *ostii* ‘Fengdan’ seedlings under different sowing date (August 30, September 15, September 30, October 15, and October 30, 2017) increased by 12.90%, 15.08%, 14.39%, 15.38% and 13.00% respectively after 3 d of soaking compared to non-soaking. Seedling height significant increased by 15.27%, 2.67%, 4.58%, 2.79% and 8.61%, leaf length significant increased by 2.92%, 2.29%, 3.68%, 2.26% and 6.84%, leaf width significant increased by 1.81%, 1.79%, 2.43%, 1.11% and 26.20%, respectively. With the increase of soaking duration to 5 d, all indexes showed a downward trend, but they were still significantly higher than those.

**Table 2:** Effect of soaking duration and sowing date on germination and seedling growth of *P*. *ostii* ‘Feng Dan’.

Sowing date	Soaking duration (d)	Germination percentage (%)	Seeding height (cm)	Leaf length (cm)	Leaf width (cm)	Leaf number
August 30, 2017	0	54.00±2.24 g	5.75±0.14 h	6.99±0.17 g	3.25±0.10 h	1.00±0.00
	3	62.00±2.74 bcd	6.07±0.17 g	7.20±0.11 def	3.31±0.11 g	1.00±0.00
	5	61.50±1.37 bcde	6.05±0.18 g	7.19±0.11 f	3.29±0.96 g	1.00±0.00
	7	54.50±4.11 fg	5.75±0.14 h	7.01±0.16 g	6.25±0.09 h	1.00±0.00
September 15, 2017	0	53.50±1.37 g	6.19±0.11 def	7.24±0.10 def	4.39±0.12 def	1.00±0.00
	3	63.00±1.12 abcd	6.36±0.13 bcd	7.41±0.12 b	4.47±0.13 bcd	1.00±0.00
	5	63.00±2.09 abcd	6.32±0.16 cdef	7.31±0.12 bcd	4.41±0.13 cdef	1.13±0.35
	7	58.00±2.09 ef	6.30±0.14 cdef	7.3±0.08 cde	4.41±0.12 cdef	1.07±0.26
September 30, 2017	0	56.50±2.85 fg	6.25±0.11 def	7.31±0.10 bcd	4.41±0.11 def	1.00±0.00
	3	66.00±3.79 a	6.55±0.17 a	7.59±0.19 a	4.52±0.10 a	1.33±0.49
	5	65.00±1.77 ab	6.40±0.11 bc	7.51±0.16 a	4.51±0.13 bc	1.20±0.41
	7	61.00±2.85 cde	6.35±0.12 bcd	7.33±0.10 bcd	4.45±0.14 bcd	1.13±0.35
October 15, 2017	0	55.00±2.50 fg	6.27±0.14 def	7.36±0.11 bc	4.44±0.11 def	1.13±0.35
	3	65.00±3.06 ab	6.45±0.18 ab	7.53±0.12 a	4.49±0.10 ab	1.13±0.35
	5	64.50±2.09 abc	6.33±0.14 bcde	7.51±0.15 a	4.49±0.14 bcde	1.20±0.41
	7	60.00±1.77 de	6.32±0.13 cdef	7.28±0.07 cdef	4.41±0.12 cdef	1.13±0.35
October 30, 2017	0	53.50±2.85 g	5.73±0.29 h	6.81±0.17 h	3.24±0.17 h	1.00±0.00
	3	61.50±2.85 bcde	6.27±0.14 def	7.31±0.10 bcd	4.39±0.13 def	1.00±0.00
	5	61.50±1.37 bcde	6.21±0.12 def	7.25±0.12 def	4.35±0.12 def	1.13±0.35
	7	54.50±4.11 fg	6.25±0.12 def	7.23±0.07 def	4.34±0.10 def	1.07±0.26

Note: Different lowercase letters indicate that the significant difference between each treatment at 0.05 level.

Except for the leaves number, the other morphological indexes showed a trend of first increasing and then decreasing In general, results indicated that soaking duration also had an impact on seedling growth (Table 2). The seed germination percentage was the highest (66.00±3.79%) at 30 September after 3 days of seed soaking. Moreover, the seedling height (6.55±0.17 cm), leaf length (7.59±0.19 cm), and leaf width (4.52±0.10 cm) were significantly higher than those of other treatments. These results indicate that both sowing time and soaking duration had an effect on germination and seedling growth in *P*. *ostii* ‘Fengdan’.

### 3.3 Effect of sowing date on rooting of *P*. *ostii* ‘Fengdan’

In the soaking test conducted in 2017, the optimum soaking duration was 3 days, so this duration of soaking was selected for use in the sowing test conducted in 2018. Results indicated that the maximum rooting percentage (94.00±1.00% and 93.33±2.52%) occurred in seeds planted on September 20, 2018 and September 30, 2018, respectively. These seeds also exhibited the shortest time to hypocotyl dormancy-release (26 days and 28 days) (Table 3).

**Table 3:** Effect of sowing date on rooting of *P*. *ostii* ‘Feng Dan’.

Sowing date	First rooting days (d)	Rooting percentage (%)	Average Air temperature (℃)		Average ground temperature within 10 days (℃)			
			Maximum Air temperature (℃)	Minimum Air temperature (℃)	5 cm	10 cm	15 cm	20 cm
September 10, 2018	30	87.67±3.21 b	24	16	23.8	24	24.2	24.2
September 20, 2018	26	94.00±1.00 a	25	16	19.8	20	20	20
September 30, 2018	28	93.33±2.52 a	23	12	15	15	16.5	16.5
October 10, 2018	36	90.67±0.58 ab	23	10	11	11.5	11.5	11.5
October 20, 2018	29	91.00±1.00 ab	18	10	15	15	15.5	15.5
October 30, 2018	37	89.00±1.00 b	23	7	11.5	12	12	12.5

The timing of hypocotyl growth varied with sowing date. Data recorded on soil temperature at the date of sowing indicated that soil temperature varied with sowing date. The best soil temperature for rooting of *P*. *ostii* ‘Fengdan’ was about 20℃, followed by 15℃, indicating that a range of 15–20℃ was favorable for breaking hypocotyl dormancy in *P*. *ostii* ‘Fengdan’ (Table 3).

### 3.4 Effect of sowing date and sowing depth on rooting of *P*. *ostii* ‘Fengdan’

**Fig 4:**
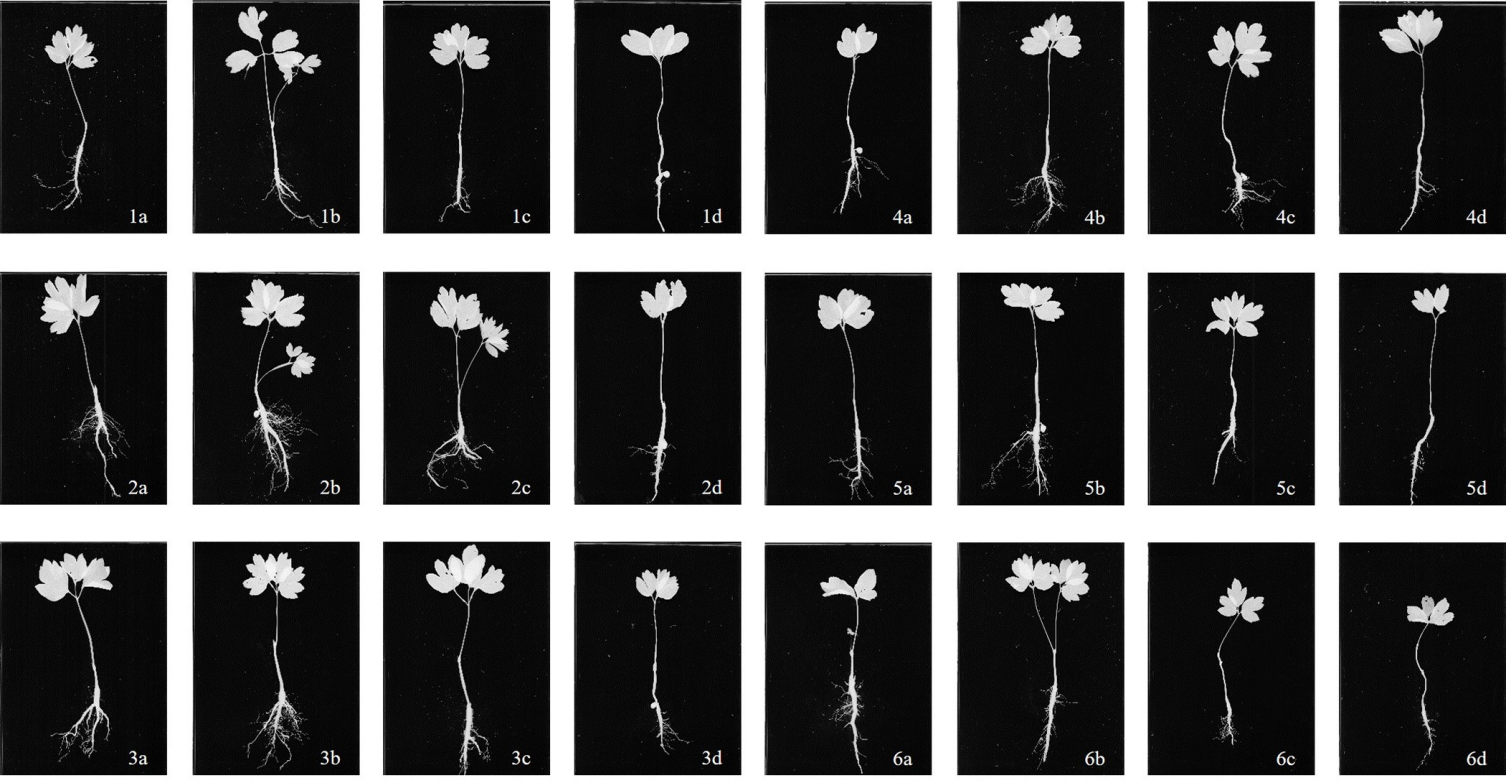
Root scanning of *Paeonia ositt* ‘Fengdan’ with different sowing duration and seeding depth. 1–6: Sowing time is September 10, September 20, September 30, October 10, October 20 and October 30; a-d: Sowing depth is 2.5 cm, 5 cm, 7.5 cm and 10 cm.

Both sowing depth and sowing date also had a significant effect on the root parameters of *P*. *ostii* ‘Fengdan’ (Table 4, Fig 4). Analysis of root length, average root diameter and the number of root tip, further confirmed the effect of different sowing times and different sowing depths on root growth. Analysis of the data indicated the following trend of September 20 > September 30 > October 10 > September 10 > October 20 > October 30, and best sowing depths showed the trend of 5 cm > 7.5 cm > 2.5 cm > 10 cm (Table 4). Both time and depth of sowing affected the growth of the root system in *P*. *ostii* ‘Fengdan’. The best rooting percentage (94.00±1.00%) was observed in seeds planted at 5 cm in depth on September 20, 2018. Compared with other sowing depth in the same period, it was significantly increased by 1.78–2.12%. The rooting percentage of seeds planted at a depth of 5 cm was best, while seeds planted at a depth of 10 cm had low percentage of rooting. The rooting percentage of seeds planted on the October 30, 2018 at a depth of 10 cm was only 88.33±0.58% (Table 4). Combining the different sowing dates, the rooting percentage of *P*. *ostii* ‘Fengdan’ planted at 5 cm compared with other sowing depths were significantly increased by 1.19% (2.5 cm), 0.98% (7.5 cm) and 1.47% (10 cm), respectively.

**Table 4:** Effect of sowing date and sowing depth on rooting of *P*. *ostii* ‘Feng Dan’.

Sowing date	Sowing depth (cm)	Rooting percentage (%)	Root length (cm)	AvgDiam (mm)	Root tips
September 10, 2018	2.5	87.33±1.53 h	53.37±19.84 efgh	17.88±1.92 abcd	120.67±36.30 ghijkl
	5	87.67±3.21 gh	86.34±17.09 bcde	19.98±2.18 ab	166.67±12.86 abcdef
	7.5	87.33±1.15 h	83.85±16.35 bcdefg	19.86±2.65 ab	165.67±12.90 abcdef
	10	87.00±0.00 h	48.17±7.62 fgh	13.05±1.93 fg	55.67±25.70 n
September 20, 2018	2.5	92.00±1.00 abcd	58.77±18.17 defgh	19.62±0.49 ab	163.00±29.87 bcdefg
	5	94.00±1.00 a	123.87±22.61 a	21.72±0.82 a	208.33±24.44 a
	7.5	92.33±1.53 abc	117.59±25.79 ab	20.75±0.69 a	188.67±11.02 abc
	10	92.00±1.00 abcd	65.22±14.88 cdefgh	15.42±3.75 cdef	90.00±11.53 jklmn
September 30, 2018	2.5	90.67±0.58 bcdef	71.94±13.33 cdefgh	19.60±0.75 ab	153.33±5.51 cdefgh
	5	93.33±2.52 ab	123.24±34.80 a	21.19±1.55 a	198.67±31.90 ab
	7.5	92.00±1.00 abcd	97.53±11.09 abc	20.66±2.19 a	177.67±17.01 abcd
	10	91.33±0.58 abcde	55.78±26.88 efgh	14.30±3.54 defg	85.00±15.40 klmn
October 10, 2018	2.5	89.33±2.08 defgh	56.29±24.07 efgh	19.60±1.93 ab	130.67±21.50 efghij
	5	90.67±0.58 bcdef	95.18±17.39 abcd	20.41±0.65 a	180.67±22.37 abcd
	7.5	89.67±0.58 cdefgh	86.78±13.93 bcde	20.37±0.17 a	173.67±25.11 abcde
	10	89.33±0.58 defgh	52.15±27.28 efgh	13.92±1.16 efg	79.00±23.52 lmn
October 20, 2018	2.5	89.00±2.00 efgh	51.33±13.71 efgh	17.65±2.20 abcde	68.67±29.14 mn
	5	91.00±1.00 bcdef	85.27±11.05 bcdef	19.14±4.56 abc	141.67±20.13 defghi
	7.5	90.33±1.53 cdefg	81.58±15.35 cdefg	18.31±2.18 abc	126.67±29.91 fghijk
	10	89.67±3.06 cdefgh	46.70±19.70 gh	12.22±1.61 fg	53.00±9.85 n
October 30, 2018	2.5	87.00±0.00 h	48.67±7.62 fgh	15.93±1.77 bcdef	60.33±33.38 mn
	5	89.00±1.00 efgh	80.95±15.89 cdefg	18.47±2.17 abc	115.00±31.75 hijkl
	7.5	88.67±1.15 efgh	72.55±11.68 cdefgh	18.22±1.18 abc	101.00±15.40 ijklm
	10	88.33±0.58 fgh	41.85±18.61 h	11.14±1.12 g	51.33±19.35 n

Note: Different lowercase letters indicate that the significant difference between each treatment at 0.05 level.

The root traits were the most optimum in seeds planted at a 5 cm depth on September 20, 2018 (Fig 4 and 2B). Under these conditions, total root length was up to 123.87±22.61 cm, and the average root diameter was 21.72±0.82 mm, which was significantly greater than in the other treatments (Table 4). The next best root parameters were observed in seeds planted at 5 cm on September 30, 2018, with a root length of 123.24±34.80 cm and an average root diameter of 21.19±1.55 mm (Fig 4 and 3B). Roots in seeds planted on October 30, 2018 at a depth of 10 cm, however, grew poorly, exhibiting a total root length of only 41.85±18.61 cm (Figs 4: 6D). These results indicate that planting seeds too deep is not conducive to root growth.

The number of root tips present was also affected by sowing time and sowing depth. The number of observed root tips in *P*. *ostii* ‘Fengdan’ ranged from 51.33±19.35 to 208.33±24.44 in the various treatments (Table 4). Similar to total root length and average root diameter, the number of root tips was maximum (208.33±24.44) when seeds were planted at a depth of 5 cm on September 20. This value was significantly greater than the number of root tips observed in the other treatments (Table 4). Analysis of the data indicated that development of the root system was inhibited when seeds were planted at a depth > 5cm and that root traits exhibited a decreasing trend in general with increasing soil depth (Table 4).

### 3.5 Effect of sowing date and sowing depth on germination and seedling growth of *P*. *ostii* ‘Fengdan’

Sowing depth had a significant effect on the growth of *P*. *ostii* ‘Fengdan’ seedlings. The percentage of seed germination increased first with planting depth, regardless of time of planting, and then decreased (Table 5). Maximum germination occurred at a seeding depth of 5 cm. All measured morphological indicators (except the number of leaves) followed the same trend as percentage germination, first increasing and then decreasing. The order of best seed performance was: 5 cm > 7.5 cm > 2.5 cm > 10 cm.

**Table 5:** Effect of sowing date and sowing depth on germination and seedling growth of *P*. *ostii* ‘Feng Dan’.

Sowing date	Seeding depth (cm)	Germination percentage (%)	Seedling height (cm)	Leaf length (cm)	Leaf width (cm)	Leaf number
September 10, 2018	2.5	52.00±29.46 bcde	13.11±0.22 i	15.47±0.43 efgh	10.02±0.33 cdefg	1.00±0.00
	5	67.00±29.10 abc	13.45±0.23 cdefgh	15.99±0.40 ab	10.17±0.26 abc	1.18±0.40
	7.5	31.00±10.15 defg	13.28±0.38 fghi	15.95±0.49 abc	10.05±0.32 cdef	1.00±0.00
	10	22.33±10.26 fgh	13.05±0.19 i	15.13±0.21 h	9.77±0.21 gh	1.00±0.00
September 20, 2018	2.5	78.33±5.03 ab	13.77±0.29 abc	15.80±0.17 abcd	10.15±0.20 abc	1.00±0.00
	5	88.33±4.04 a	14.07±0.34 a	16.16±0.47 a	10.38±0.26 a	1.45±0.69
	7.5	56.00±4.36 bcd	13.85±0.53 ab	15.98±0.48 abc	10.25±0.30 abc	1.09±0.30
	10	53.00±20.66 bcde	13.80±0.29 ab	15.63±0.26 cde	10.14±0.21 bc	1.00±0.00
September 30, 2018	2.5	63.33±12.70 abc	13.64±0.39 bcde	15.73±0.18 bcd	10.11±0.17 bcd	1.00±0.00
	5	79.00±4.58 ab	14.01±0.28 a	15.94±0.30 abc	10.33±0.25 ab	1.18±0.40
	7.5	52.00±2.00 bcde	13.67±0.53 bcd	15.81±0.53 bcd	10.22±0.33 abc	1.09±0.30
	10	49.33±20.79 cde	13.60±0.42 bcdef	15.36±0.21 efgh	9.99±0.16 cdefg	1.00±0.00
October 10, 2018	2.5	56.00±3.00 bcd	13.35±0.28 defghi	15.53±0.34 defg	10.05±0.18 cdef	1.00±0.00
	5	62.67±8.50 abc	13.78±0.38 abc	15.63±0.29 cde	10.22±0.28 abc	1.09±0.30
	7.5	26.33±18.15 efgh	13.50±0.62 cdefgh	15.60±0.38 def	10.13±0.34 bcde	1.00±0.00
	10	20.00±17.32 fgh	13.22±0.20 ghi	15.19±0.23 gh	9.86±0.18 defgh	1.00±0.00
October 20, 2018	2.5	55.33±11.02 bcd	13.21±0.13 ghi	15.21±0.25 fgh	9.79±0.18 fgh	1.00±0.00
	5	60.00±18.25 bc	13.55±0.25 bcdefg	15.40±0.36 efgh	10.08±0.19 bcde	1.00±0.00
	7.5	11.00±7.94 gh	13.33±0.49 efghi	15.35±0.25 efgh	10.02±0.39 cdefg	1.00±0.00
	10	9.33±8.62 gh	13.07±0.16 i	14.48±0.49 i	9.50±0.28 i	1.00±0.00
October 30, 2018	2.5	18.00±14.93 fgh	13.04±0.19 i	15.15±0.12 h	9.66±0.18 hi	1.00±0.00
	5	42.33±17.04 cdef	13.15±0.13 hi	15.26±0.28 fgh	10.01±0.20 cdefg	1.00±0.00
	7.5	9.67±6.43 gh	13.09±0.43 i	15.22±0.19 gh	9.85±0.26 efgh	1.00±0.00
	10	3.33±2.52 h	13.03±0.18 i	14.45±0.44 i	9.25±0.32 j	1.00±0.00

Note: Different lowercase letters indicate that the significant difference between each treatment at 0.05 level.

The analysis on the combined effect of sowing depth and time of sowing on seedling performance revealed that seeds planted at a depth of 5 cm on September 20 had the best growth, exhibiting a germination percentage of 88.33±4.04%, an average seedling height of 14.07±0.34 cm, an average leaf length of 16.16±0.47 cm, and an average leaf width of 10.38±0.26 cm. The lowest growth measurements were observed in seeds planted on October 30 at a planting depth of 10 cm, where the germination percentage was only 3.33±2.52%, the average seedling height was 13.03±0.18 cm, the average leaf length was 14.45±0.44 cm, and the average leaf width was 9.25±0.32 cm (Table 5). These results were consistent with the results obtained on root growth.

### 3.6 Effect of the exogeneous application of different compounds on germination and seedling growth in *P*. *ostii* ‘Fengdan’

The germination percentage of *P*. *ostii* ‘Fengdan’ seeds treated with 5-ALA was significantly higher (*P* < 0.05) than that of the control group. Notably, the effects of different concentration of 5-ALA varied with soaking duration (Table 6). Results revealed a tendency for low concentration of 5-ALA to promote seed germination and growth, while high concentration of 5-ALA inhibited both seed germination and growth. Germination percentage and morphological indices increased with increased soaking period in treated with 0.38 mmol/L 5-ALA. The results showed that at low concentrations (0.76 mmol/L and 1.52 mmol/L), the germination percentage, seedling height, leaf length and leaf width increased first and then decreased with the increase of soaking duration. When the concentration of 5-ALA increased to 3.04 mmol/L, the seed germination percentage and seedling growth indexes gradually decreased with the extension of soaking duration. The seeds soaked in 0.76 mmol/L 5-ALA for 48 h had the greatest beneficial impact on seedling growth, with germination percentage of 78.67±4.73%, average seedling height of 11.55±0.47 cm, average leaf length of 15.81±0.55 cm, and average leaf width of 9.84±0.48 cm (Table 6). The germination percentage of *P*. *ostii* ‘Fengdan’ treated with 0.76 mmol/L 5-ALA for 48 h was significantly increased by 4.25% (24 h) and 5.08% (72 h) compared with other treatments. The collective results indicate that 5-ALA has a dual effect on the germination and growth of *P*. *ostii* ‘Fengdan’.

**Table 6:** Effect of different concentrations and soaking duration of 5-ALA on germination and seedling growth of *P*. *ositt* ‘Fengdan’ seeds.

Concentration	Soaking duration (h)	Germination percentage (%)	Seeding height (cm)	Leaf length (cm)	Leaf width (cm)	Leaf number
CK	24	61.00±1.00 e	10.00±0.35 g	13.75±0.64 f	8.33±0.48 e	1.00±0.00
	48	63.33±3.21 de	10.03±0.38 g	13.93±0.63 f	8.34±0.56 e	1.07±0.26
	72	64.67±3.79 cde	10.09±0.34 fg	14.06±0.39 ef	8.53±0.47 de	1.07±0.26
0.38 mmol/L ALA	24	69.33±0.58 bcd	10.13±0.51 fg	14.60±0.48 bcd	8.93±0.46 bcd	1.00±0.00
	48	71.33±2.52 abc	10.39±0.47 efg	14.74±0.50 b	9.14±0.24 bc	1.00±0.00
	72	73.67±1.53 ab	10.49±0.52 def	14.95±0.34 b	9.16±0.63 bc	1.00±0.00
0.76 mmol/L ALA	24	75.33±6.11 ab	11.16±0.47 a	15.27±0.55 a	9.75±0.56 a	1.20±0.41
	48	78.67±4.73 a	11.55±0.47 a	15.81±0.55 a	9.84±0.48 a	1.27±0.46
	72	74.67±2.08 ab	10.82±0.92 b	14.95±0.79 b	9.18±0.48 bc	1.13±0.35
1.52 mmol/L ALA	24	73.67±3.51 ab	11.01±0.78 b	15.05±0.51 b	9.31±0.32 b	1.13±0.35
	48	73.67±6.81 ab	10.82±0.53 b	15.04±0.48 b	9.27±0.54 b	1.07±0.26
	72	71.33±2.52 abc	10.64±0.44 b	14.83±0.53 b	9.17±0.63 bc	1.00±0.00
3.04 mmol/L ALA	24	73.00±3.61 ab	10.25±0.44 bc	14.81±0.60 bc	9.06±0.31 bc	1.00±0.00
	48	71.33±9.07 abc	10.17±0.41 fg	14.60±0.60 cde	8.77±0.40 bcde	1.00±0.00
	72	69.33±1.15 bcd	10.13±0.38 fg	14.59±0.44 def	8.67±0.34 cde	1.00±0.00

Note: Different lowercase letters indicate that the significant difference between each treatment at 0.05 level.

This experiment observed that the germination behavior of *P*. *ostii* ‘Fengdan’ seeds treated with different concentrations of SNP was similar to that treated with 5-ALA (Table 7). All of the SNP treatments were found better than the control group with regard to its effect on germination. The germination percentage and morphological parameters of seedlings increased with increasing soaking duration (24 h-72 h) in seeds treated with 5 mmol/L SNP. When the SNP concentration was increased to 10 mmol/L and 15 mmol/L, the germination percentage, seedling height, leaf length, and leaf width first increased and then decreased with increasing soaking duration. When the concentration of SNP was increased to 20 mmol/L, the seed germination percentage and seedling growth exhibited a gradually decreasing trend with increasing soaking duration. The results showed that 10 mmol/L SNP was the optimal treatment concentration, and the effect of soaking for 48 h significantly reached the best. Under this treatment, the germination percentage of seeds was 76.67±11.24% and the average seedling height was 13.50±0.44 cm, the average leaf length was 16.40±0.27 cm, and the average leaf width was 9.87±0.23 cm (Table 7). The germination percentage of *P*. *ostii* ‘Fengdan’ treated with 10 mmol/L SNP for 48 h was significantly increased by 4.36% (24 h) and 7.40% (72 h) compared with other treatments.

**Table 7:** Effect of different concentrations and soaking duration of SNP on germination and seedling growth of *P*. *ositt* ‘Fengdan’ seeds.

Concentration	Soaking duration (h)	Germination percentage (%)	Seeding height (cm)	Leaf length (cm)	Leaf width (cm)	Leaf number
CK	24	61.00±1.00 e	10.00±0.35 f	13.75±0.64 f	8.33±0.48 d	1.00±0.00
	48	63.33±3.21 de	10.03±0.38 ef	13.93±0.63 f	8.34±0.56 d	1.07±0.26
	72	64.67±3.79 cde	10.09±0.34 ef	14.06±0.39 f	8.53±0.47 d	1.07±0.26
5 mmol/L SNP	24	69.00±2.00 abcd	10.97±0.43 d	15.66±0.43 c	9.33±0.40 b	1.07±0.26
	48	70.33±1.53 abcd	10.99±0.51 d	15.82±0.53 bc	9.41±0.29 b	1.13±0.35
	72	70.67±1.53 abcd	11.47±0.24 c	15.85±0.36 bc	9.45±0.26 b	1.13±0.35
10 mmol/L SNP	24	73.33±0.58 ab	12.57±0.42 b	16.34±0.37 a	9.87±0.42 a	1.33±0.49
	48	76.67±11.24 a	13.50±0.44 a	16.40±0.27 a	9.87±0.23 a	1.20±0.41
	72	71.00±4.58 abcd	11.52±0.36 c	16.05±0.39 ab	9.52±0.43 b	1.13±0.35
15 mmol/L SNP	24	70.33±1.53 abcd	11.51±0.45 c	15.89±0.40 bc	9.55±0.35 b	1.13±0.35
	48	73.00±4.58 abc	11.75±0.43 c	16.07±0.47 ab	9.85±0.43 a	1.20±0.41
	72	69.67±7.23 abcd	11.45±0.42 c	15.87±0.48 bc	9.47±0.21 b	1.13±0.35
20 mmol/L SNP	24	69.67±2.52 abcd	10.95±0.52 d	15.27±0.43 d	9.34±0.23 b	1.07±0.26
	48	66.67±2.08 bcde	10.35±0.37 e	14.95±0.54 de	9.29±0.47 b	1.07±0.26
	72	65.67±2.08 bcde	10.11±0.33 ef	14.83±0.54 e	8.90±0.26 c	1.00±0.00

Note: Different lowercase letters indicate that the significant difference between each treatment at 0.05 level.

### 3.7 Effect of different exogenous substances on several physiological characteristics of *P*. *ostii* ‘Fengdan’

**Fig 5:**
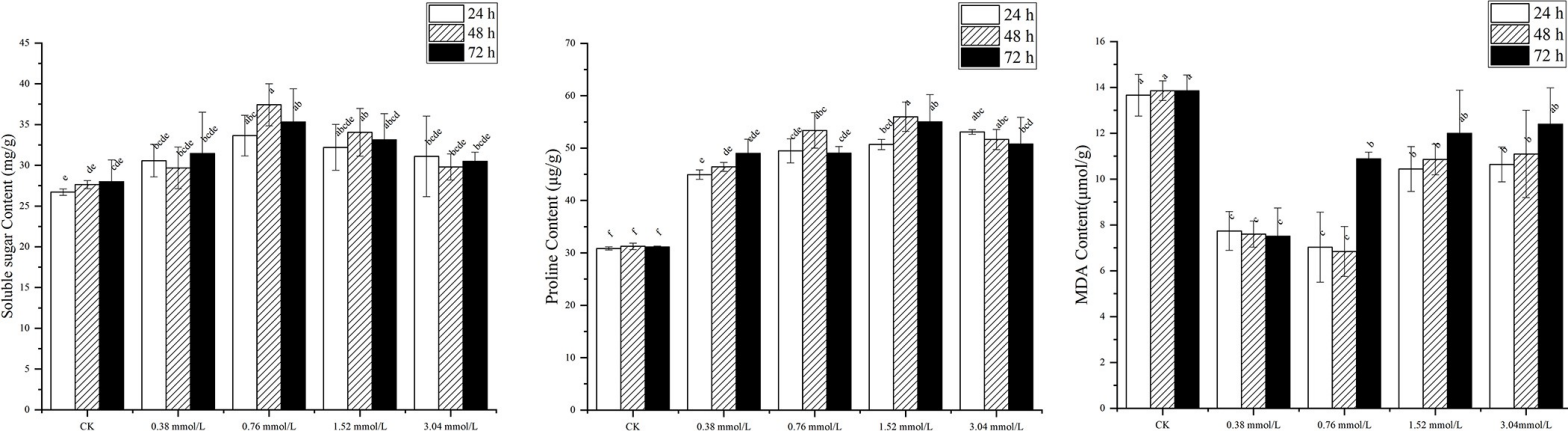
Soluble sugar content (A), proline content (B), and MDA content (C) in leaves of *P*. *ositt* ‘Fengdan’ seedlings after soaking with different concentrations of 5-ALA.

Further analyses revealed that the 5-ALA treatments also enhanced level of total soluble sugar in *P*. *ostii* ‘Fengdan’ seedlings, with the variation pattern in soluble sugar levels being similar to the seedling morphological indices (Fig 5A). The maximum soluble sugar content in leaves was obtained by soaking seeds in 0.76 mmol/L 5-ALA for 48 h. Soluble sugars in leaves of seedlings whose seeds were soaked at 24 h in 0.38 mmol/L, 0.76 mmol/L, 1.52 mmol/L, or 3.04 mmol/L 5-ALA increased significantly by 14.46%, 25.96%, 20.56% and 16.41%, relative to the control, respectively. Soluble sugars in leaves of seedlings whose seeds were soaked in 0.38 mmol/L, 0.76 mmol/L, 1.52 mmol/L, or 3.04 mmol/L 5-ALA for 48 h increased significantly by 7.41%, 35.46%, 23.24% and 7.82%, relative to the control, respectively. While the 72 h immersion of seeds in the same concentrations of 5-ALA increased significantly soluble sugar content in seedlings by 12.39%, 26.20%, 18.36% and 8.97%, relative to the control, respectively.

The proline content in leaves of seedlings derived from seeds treated with 5-ALA was higher than it was in the water control (Fig 5B). The maximum content of proline was observed in leaves of seedlings derived from seeds treated with 1.52 mmol/L 5-ALA for 48 h, resulting in a 79.12% increase, relative to the control group. Results also indicated that the MDA content in leaves was also lower in the 5-ALA seed treatment (Fig 5C). The MDA content was lowest in leaves of seedlings derived from seeds treated with 0.76 mmol/L 5-ALA for 48 h.

**Fig 6:**
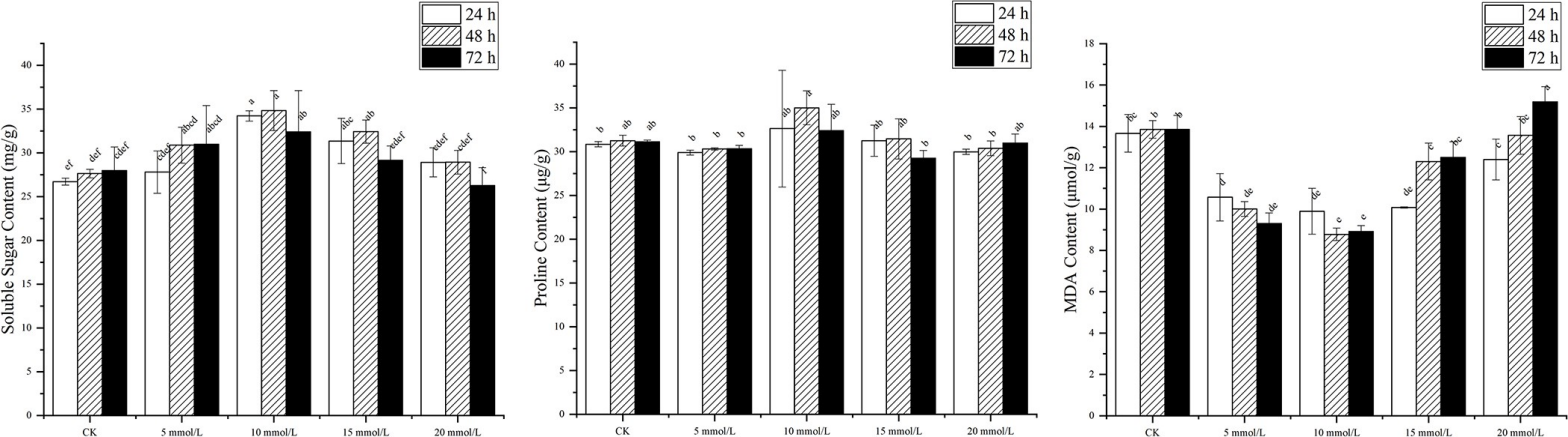
Soluble sugar content (A), proline content (B), and MDA content (C) in leaves of *P*. *ositt* ‘Fengdan’ seedlings after soaking with different concentrations of SNP.

The levels of soluble sugars, proline, and MDA in leaves of seedling derived from SNP-treated seeds varied with the duration of seed soaking (Fig 6). Seeds treated with 5 mmol/L SNP showed an increasing trend in the level of soluble sugar in leaves with increasing duration of seed soaking. The content of soluble sugar in seedlings leaves treated with 10 mmol/L and 15 mmol/L exhibited an increasing trend followed by a decrease. However, the soluble sugar level decreased under 20 mmol/L treatment. Soaking seeds in a 10 mmol/L SNP solution for 48 h increased the level of soluble sugar in seedling leaves by 26.01%, compared to the same soaking duration in the water control (Fig 6A). The proline content of 10 mmol/L SNP increased by 5.81% (24 h), 12.02% (48 h) and 4.08% (72 h) with the increase of soaking duration, respectively, compared to that of water treatment, and the increase of proline content in *P*. *ostii* ‘Fengdan’ seedling leaves was the largest at 48 h (Fig 6B). The 10 mmol/L SNP treatment for 48 h was found to significantly reduce MDA content in leaves of seedlings derived from treated seeds by 36.67% compared with the control. After soaking seeds with 20 mmol/L SNP for 72 h, MDA content in seedling leaves reached the maximum (15.19±0.73 μmol/g), which was significantly higher than that in control (9.67%) (Fig 6C).

## 4 Discussion

Germination of *P*. *ostii* ‘Fengdan’ seeds generally requires 8–9 months under natural conditions. The main problem of *P*. *ostii* ‘Fengdan’ production is seed dormancy [34–36]. Dormancy breaking in *P*. *ostii* ‘Fengdan’ seeds involves two processes which are dormancy release in hypocotyls and epicotyls, respectively. The timing and conditions for dormancy-release in epicotyl and hypocotyls are different. Warm environment conditions are required for radicle growth followed by cold temperatures to break epicotyl germinate [37, 38]. The epicotyl dormancy in tree peony seeds cannot be broken until roots have grown longer than 3 cm [39, 40]. This study found that taproot length > 50 mm are only required for seed germination, which is consistent with results reported by Ren et al. [41]. A previous study reported that post-ripening of the embryo is completed in *P*. *ostii* ‘Fengdan’ when root length reaches 4 cm, at which time the embryo is then receptive to low-temperature for completion of vernalization. When the length of roots exceeds 6–7 cm, the nutrient content in the embryo decreases sharply, indicating that high levels of nutrients are no longer required by the epicotyl [42]. The results of the present study indicate, however that a root length of > 5 cm is a sufficient marker for this stage of embryo maturity.

Most studies have focused on the release of epicotyl dormancy by low temperature and chemical treatment, rather than the release of hypocotyl dormancy [43]. This study indicates that too short soaking time, insufficient water absorption occurs, and the seed coat remains hard, which is not conducive to hypocotyl growth, affect seed germination. While soaking duration was too long, seeds were easily rotted and anaerobic respiration was induced due to insufficient oxygen, resulting in a low emergence percentage. Therefore, immersion of seeds in water at room temperature for 3 days is sufficient to break hypocotyl dormancy and facilitate seed germination.

Temperature is an important factor affecting germination percentage and duration, as well as the level of dormancy acquired during seed maturation [44]. The temperature of 15–20℃ is sufficient to break hypocotyl dormancy in *P*. *ostii* ‘Fengdan’ seeds, which is similar to the results reported by Chen *et al*. [45]. Due to the variations in meteorological conditions that exist year to year, measuring soil temperature should be the most direct method to determine whether the conditions are suitable for breaking hypocotyl dormancy in *P*. *ostii* ‘Fengdan’ seeds. The current experiments provided important information on the dormancy of the hypocotyl axis of *P*. *ostii* ‘Fengdan’ seeds. The root system absorbs water and nutrients from the soil that can be utilized for the growth of the whole plant [46]. This study found that increasing the depth of planting limited the growth of the root system in *P*. *ostii* ‘Fengdan’. The root length in a unit soil volume is an important indicator for evaluating the ability of roots to absorb water and nutrients [47]. When root growth space is limited, the spatial distribution of roots in a given soil volume plays an important role in the absorption of water and nutrients, which in turn impacts plant growth [48, 49].

5-ALA is an essential precursor for the biosynthesis of tetrapyrrols in biological systems, which is ubiquitous in plants [50], and can also be chemically synthesized. 5-ALA not only plays the regulatory role in seed germination [51, 52] and photosynthesis [53, 54] in crops, but also alleviates abiotic stresses [55, 56]. Exogenous 5-ALA has been applied to plant to study its effect on stress response in plants [57, 58]. In the present study, the effect of 5-ALA was found to depend on concentrations. With the increase of soaking duration, the effects of 0.76 and 1.52 mmol/L 5-ALA on seed germination percentage, seedling height, leaf length and leaf width, soluble sugar and proline in leaves of *P*. *ostii* ‘Fengdan’ increased first and then decreased. The results showed that the growth of *P*. *ostii* ‘Fengdan’ was sensitive to the change of 5-ALA concentration. It is consistent with some previous evidence that high concentrations of 5-ALA inhibit plant growth while low concentrations promote it [59, 60]. SNP is a standard source of exogenous NO, and Delledonne *et al*. [61] demonstrated that 0.5 mmol/L SNP can produce 2.0 μmol/L of NO when exposed to water. Nitric oxide released by the exogenous application of SNP has been shown to have significant physiological effects on seed dormancy and seed germination [62]. As shown in this experiment, the germination percentage, plant biomass, soluble sugar content, and proline content were also higher in seedlings of *P*. *ostii* ‘Fengdan’ derived from the treatment with lower concentrations of SNP, relative to the untreated control. Higher concentrations of these compounds and increased duration of soaking of seeds, however, had an adverse effect on germination and seedling growth parameters. Comparing with previous experiments, the same results were observed in this study, that is, 5-ALA and SNP have similar action characteristics as plant hormones [63, 64].

Soluble sugar and proline are important osmotic regulatory substances in plants, which can improve the ability of cells to retain water [65]. This study showed that 0.76 mmol/L 5-ALA and 10 mmol/L SNP treatments could significantly increase the contents of soluble sugar and proline in *P*. *ostii* ‘Fengdan’ seedlings, indicating that these two treatments had favorable effects on seedling growth. These results were not unexpected, as previous reports have demonstrated that 5-ALA [66] and SNP [67] at appropriate concentrations can promote plant growth by inducing physiological and biochemical changes in plants. The results of this experiment thus further implying their importance. MDA content can be used as one of the indexes of free radical toxicity of plants [68]. This study found that soaking seeds in appropriate concentrations of 5-ALA or SNP can also reduce MDA levels in the leaves of seedlings derived from the treated seeds. These results are consistent with the previously reported beneficial effect of 5-ALA [69] and SNP [70] on reducing the negative impact of environmental stress in plants.

## 5. Conclusion

The effects of various natural measures on rooting, seedling germination, root development, plant growth and physiological characteristics of newly harvested leaves were studied by using the seeds of *Paeonia ostii* ‘Fengdan’ harvested in the same year. The results showed that soaking seeds in water for 3 days was beneficial to break the hypocotyl dormancy. The latter was released 26 days earlier than that of normal sowing after sand storage treatment, and the taproot length reached 50 mm being favorable for the hypocotyl dormancy removal. When the sowing depth was 5 cm and the ground temperature reached 20℃, it was most favorable for the seed germination and seedling growth of *Paeonia ostii* ‘Fengdan’. After 0.76 mmol/L 5-ALA soaking for 48 h or 10 mmol/L SNP soaking for 48 h, the seedlings of *Paeonia ostii* ‘Fengdan’ had the best growth and the highest germination rate. These results provided a new method for effectively improving the seed germination of *Paeonia ostii* ‘Fengdan’ in natural state. At the same time, it also provides technical support for high yield and high efficiency cultivation of tree peony and popularization of oil tree peony industrialization system.

## Supporting information

S1 Data (XLSX)
